# Low Dose Prenatal Ethanol Exposure Induces Anxiety-Like Behaviour and Alters Dendritic Morphology in the Basolateral Amygdala of Rat Offspring

**DOI:** 10.1371/journal.pone.0054924

**Published:** 2013-01-30

**Authors:** Carlie L. Cullen, Thomas H. J. Burne, Nickolas A. Lavidis, Karen M. Moritz

**Affiliations:** 1 School of Biomedical Sciences, The University of Queensland, St. Lucia, Queensland, Australia; 2 Queensland Brain Institute, The University of Queensland, St. Lucia, Queensland, Australia; Chiba University Center for Forensic Mental Health, Japan

## Abstract

Prenatal exposure to high levels of alcohol is strongly associated with poor cognitive outcomes particularly in relation to learning and memory. It is also becoming more evident that anxiety disorders and anxiety-like behaviour can be associated with prenatal alcohol exposure. This study used a rat model to determine if prenatal exposure to a relatively small amount of alcohol would result in anxiety-like behaviour and to determine if this was associated with morphological changes in the basolateral amygdala. Pregnant Sprague Dawley rats were fed a liquid diet containing either no alcohol (Control) or 6% (vol/vol) ethanol (EtOH) throughout gestation. Male and Female offspring underwent behavioural testing at 8 months (Adult) or 15 months (Aged) of age. Rats were perfusion fixed and brains were collected at the end of behavioural testing for morphological analysis of pyramidal neuron number and dendritic morphology within the basolateral amygdala. EtOH exposed offspring displayed anxiety-like behaviour in the elevated plus maze, holeboard and emergence tests. Although sexually dimorphic behaviour was apparent, sex did not impact anxiety-like behaviour induced by prenatal alcohol exposure. This increase in anxiety – like behaviour could not be attributed to a change in pyramidal cell number within the BLA but rather was associated with an increase in dendritic spines along the apical dendrite which is indicative of an increase in synaptic connectivity and activity within these neurons. This study is the first to link increases in anxiety like behaviour to structural changes within the basolateral amygdala in a model of prenatal ethanol exposure. In addition, this study has shown that exposure to even a relatively small amount of alcohol during development leads to long term alterations in anxiety-like behaviour.

## Introduction

Consumption of alcohol during pregnancy can be detrimental to the healthy development of the fetus as well as continued growth and development after birth. The fetal nervous system exhibits a particular vulnerability to alcohol induced damage [1], [2]. Consumption of large amounts of alcohol during pregnancy often results in a specific pattern of facial, cranial and cerebral malformations and mental retardation in the offspring known as fetal alcohol syndrome (FAS) [3], [4], [5], [6], [7], [8]. FAS is the most severe outcome in a broad spectrum of cognitive and behavioural effects associated with fetal alcohol exposure collectively grouped under the umbrella term fetal alcohol spectrum disorders (FASD) [9], [10], [11], [12]. These poor cognitive outcomes can include difficulties with learning and memory [4], [13], [14], [15], [16], [17], [18], [19], social interaction and engagement [20], [21], hyperactivity and attention [4], [10], [20]. Many of these problems are most prominent during schooling but can often persist well into and throughout adulthood [4], [22], [23], [24].

While both clinical and experimental research has largely focussed on learning, memory and attention difficulties, emerging clinical studies suggest a strong correlation between prenatal alcohol exposure and the incidence of anxiety related disorders during adolescence and adulthood [22], [23]. Further to this, Famy et al [22] reported a strong sex effect in the incidence of anxiety disorders in a group of 25 adults with FAS or fetal alcohol effects (FAE), with 50% of women being diagnosed with an anxiety disorder compared to nil occurrences in men. Famy et al [22] also reported that 19% of subjects exposed to prenatal alcohol were diagnosed with Antisocial Personality Disorder and 29% diagnosed with Avoidant Personality Disorder.

Despite the clinical evidence suggesting anxiety and antisocial behaviour as a potential long term problem relating to prenatal alcohol exposure, only a handful of experimental studies have investigated this relationship. However, the majority of these studies report an anxiety-like phenotype as a result of high levels of prenatal alcohol exposure [25], [26], [27], [28], [29], [30], [31], [32]. Dursun *et al* [27] reported a significant decrease in open arm exploration during an elevated plus maze trial in young adult male rats born from dams given 6 g/kg ethanol daily via oral gavage from gestational day (GD) 7 to GD20. In contrast, Osborne *et al* [30]found that feeding dams a liquid diet containing 36% ethanol derived calories for the duration of gestation did not induce anxiety-like behaviour in offspring of a similar age. Interestingly, sexually dimorphic behaviour in prenatal ethanol exposed animals has also been reported with female offspring showing greater anxiety-like behaviour than male offspring during an elevated plus maze test [30]. Although the timing of alcohol exposure in these studies differed, both used high dose prenatal alcohol exposure (in which peak blood alcohol was generally >0.1%) in young adult rats. To date, neither clinical nor experimental research has investigated the effect of prenatal alcohol exposure on anxiety or anxiety-like behaviour following low dose alcohol consumption during gestation. However, this is important as the majority of women that continue to drink during pregnancy, do so at moderate levels [33].

The regulation of anxiety like behaviour has been associated with the basolateral amygdala (BLA) [34]. The BLA is just one of the diverse nuclei within the amygdaloid complex which is an integral brain structure of the limbic system [35]. The BLA receives input from cortical and subcortical regions of the brain including the medial amygdaloid nucleus (MeA) and integrates the signals, then projects the information to the central nucleus of the amygdala (CeA) as well as other brain regions, which ultimately results in the expression of anxiety-like or fearful behavioural responses [36], [37]. Despite the importance of the BLA in the governance of anxiety-like behaviour, no studies have looked at the effect of prenatal alcohol exposure on the structure of this region. Our laboratory has recently established a rat model of low dose exposure to ethanol throughout pregnancy (blood alcohol reaching only 0.03%) and reported subtle effects on growth and development [38]. This study aimed to use this model to investigate the effect of chronic low dose ethanol exposure on anxiety-like behaviour as well as structural morphology within the BLA in both adult and aged offspring.

## Materials and Methods

### Ethics Statement

All animal procedures were approved by the Animal Welfare Unit of The University of Queensland and follow the code of practice for the care and use of animals for scientific purposes.

### Animals and Housing

All animals were housed under temperature and humidity controlled conditions under a 12 hr light cycle (lights on at 12∶00). Female nulliparous Sprague Dawley (University of Queensland Biological Resources, St. Lucia, QLD, AUS) rats were time mated from 8 weeks of age. At least one week prior to mating the non-pregnant females were trialled on a liquid diet modelled from the commercially available Leiber DeCarli diet [39]. The morning after mating the presence of seminal plugs indicated a successful mating and the day was marked as embryonic day (E) 1. The pregnant females were then housed singly and randomly assigned to receive a liquid diet containing 6% (vol/vol) ethanol, 15% ethanol derived calories (EDC) (n = 15), or an isocaloric control diet (n = 16) *ad libitum* for 21 hours a day for the duration of pregnancy (E1–22/23) with fresh diet prepared daily. The dams were offered water for the remaining 3 hours a day (09∶00–12∶00) and water consumption over this period of time was recorded. The dams were weighed daily at the time of diet removal but were otherwise left undisturbed. At parturition the experimental diet was removed and the dams were given *ad libitum* access to standard rat chow and water. Our laboratory has previously reported that *ad libitum* consumption of this diet by pregnant dams results in a peak plasma ethanol concentration (PEC) of 0.03±0.01% (30 mg/dl) approximately 30 minutes after offering fresh diet [38].

### Offspring

The rat pups were weighed every 3 days from postnatal day (PN) 1 to weaning at PN28. After weaning the offspring were separated by sex and housed in litter mate groups of up to 4 animals per cage with *ad libitum* access to standard rat chow and water. The offspring were weighed and had their cage changed weekly but were otherwise left undisturbed until the desired aged for testing. Data for offspring growth has been previously reported [38]. Animals were tested in one of two age groups, 7–10 months (Adult) or 15–18 months (Aged). To minimise any effect of litter bias no more than two males or two female littermates were used at any age. The number of animals included in the analysis for individual tests are indicated in the figure legends.

#### Behavioural testing

All behavioural testing was carried out during the dark phase. The animals were moved to the behavioural testing room in their home cages one hour after the light cycle change and allowed to habituate to the room for one hour prior to commencement of testing. Dim lights (∼20 lux) and a small colour camera were situated above the arena. All trials were video recorded and animal movement tracked using automated software (Ethovision ver. 5, Noldus, Netherlands). All equipment used for testing was wiped clean with 70% EtOH between trials to eliminate odour cues unless otherwise stated.

### Elevated Plus Maze (EPM)

The EPM was carried out as described in Burne et al [40]. In brief, each rat was placed in the centre of a grey acrylic maze consisting of two open (50 cm×7 cm) and two closed (50 cm×7 cm×30 cm) arms mounted on a metal stand 60 cm high, facing an open arm and left to explore the maze for a single 10 minute trial. Percentage time spent on the open arms was recorded as an indication of anxiety like behaviour.

### Holeboard (HB) Test

The HB test was carried out using a grey acrylic open field arena (60 cm×60 cm×30 cm) with a raised floor insert (5 cm above the floor) with four holes (5 cm diameter) situated 10 cm in from each corner. Each rat was placed in one corner facing the wall and left to explore the arena for 10 minutes as described in Burne et al [40]. The frequency of and time spent head dipping into the holes was recorded as a measure of neophobia. The percentage time spent in the centre of the arena was recorded as a measure of anxiety like behaviour. The total distance travelled within the arena during the 10 minute trial was also recorded as an indicator of hyperactivity.

### Social Interaction (SI) Test

The SI test was adapted from the design outlined in Burne et al [40]. One “subject” rat and a pair rat matched for age, weight, sex and treatment condition were placed at opposite ends of a novel black plastic arena (100 cm×50 cm×40 cm) facing the corner and left to explore for 10 minutes. The amount of time the “subject” rat spent sniffing/exploring the other rat was recorded as a measure of social behaviour. Rearing behaviour was recorded as an indicator of investigation of the environment.

### Emergence Test

The Emergence test was carried out in a black plastic arena (100 cm×50 cm×40 cm) with an enclosed black plexiglass box (20 cm×40 cm×30 cm) placed at one end with an open entrance (10 cm×15 cm) facing out into the remaining open area of the arena. Similar to Morely et al [41] and Baker et al [42] the rat was placed inside the dark box at the beginning of a 10 minute trial. Emergence from the dark box was deemed when head and forelimbs of the rat were clearly visible outside of the box. The latency of first emergence and time spent in the open arena were recorded as measures of anxiety like behaviour.

### Tissue Collection

At the completion of behavioural testing rats were euthanized with an overdose of sodium pentobarbital (Lethabarb; 0.1 ml/kg bodyweight) and subsequently flushed with heparinised 0.2 M Phosphate Buffered Saline (PBS) and fixed with 4% paraformaldehyde (PFA) (Sigma Aldrich) in PBS solution via intracardiac perfusion. The brains were then collected and further fixed in 4% PFA for 24 hours then stored in 0.4% PFA until histological processing.

### Histology

#### Stereology

50 µm coronal section were cut from n = 5 brains per sex, per treatment from both age groups using a Vibratome. Slices were mounted onto gelatine coated slides and Nissl stained using 0.1% Cresyl Violet. Stereological analysis was performed using StereoInvestigator (ver. 9, Mbf Biosciences) software. Pyramidal cells were distinguished from Glial cells by their large size and presence of a nucleus. Pyramidal cell number within the BLA was determined by the optical fractionator method [43]. Pyramidal neurons were counted in a total of six slices with a three slice interval between. BLA volume was determined using the Cavalieri estimator. Numerical cell density was calculated per 1000 µm^3^ (total # cells/BLA volume*1000).

### Dendritic Morphology

Brains (n = 5 per sex, per treatment) from both age groups were stained using a modified Golgi Cox staining protocol. In brief, the fixed brains were soaked in increasing concentrations (10%, 20%, 30%) of sucrose (in PBS) solution at pH 7.4 for 24hours each. The hemispheres were then separated and blocked in 4% agarose gel which was then trimmed to ensure an even distribution of gel (approx. 4–6 mm) surrounded the tissue. The brain blocks were then submerged in Golgi Cox Solution (5% Potassium dichromate, 5% Potassium chromate, 5% Mercuric chloride) and kept in the dark for 33 days changing the solution weekly. The Golgi impregnated brains were then removed and coronally sectioned at 300 µm. Slices were processed as per [44] and mounted on gelatine coated glass slides using depex mounting medium and stored in the dark.

Apical dendrites for six pyramidal cells per animal, per region were traced using NeuroLucida (ver. 9, Mbf Biosciences) and branching morphology determined using branched structure analysis. Neurons within a region were randomly selected and assessed to determine if tracing criteria were met. Cells were selected for tracing if there was a clearly visible cell soma, a well impregnated apical dendrite and dendritic spines were visible. Dendritic spines were counted along the apical dendrite and spine density was determined by dividing the number of spines counted by the total length of the apical dendrite tree.

### Statistical Analysis

All data analysis was performed using Statistical Package for the Social Sciences (ver. 17 SPSS Inc., IL, USA). Maternal data and offspring body weight was analysed using unpaired t tests. Behavioural and histological data was analysed using three-way ANOVA with Treatment, Sex and Age as factors. Post hoc analysis with a Bonferroni correction was used where significant interactions between factors were observed.

## Results

### Maternal Data

In order to assess variation in diet intake and pregnancy variables, diet consumption and maternal variables were recorded. These results are shown in Table 1. There were no significant differences in maternal diet consumption, weight gain, gestation length or litter size between dams fed an ethanol containing diet (EtOH) and dams fed an isocaloric control diet (Control).

**Table 1:** Maternal variables during gestation and offspring bodyweights at the commencement of behavioural testing.

Variable	Control	EtOH
***Maternal parameters:***		
Diet consumption	30.68±0.81 ml/day	28.36±0.83 ml/day
Total weight gain	97.4±5.8 g	97.5±6.0 g
Gestation length∧	6, 22 days; 10, 23 days	6, 22 days; 9, 23 days
Litter size	11±1	10±1
***Offspring parameters:***		
Body weight (8 months)		
Male	502.8±21.07 g	485.1±14.43 g
Female	314.6±27.95 g	292.4±7.52 g
Body weight (15 months)		
Male	594.1±13.40 g	549.7±13.14 g*
Female	334.4±13.16 g	343.7±8.15 g

∧ reported as number of occurrences.

All other data is represented as mean±SEM.

* indicates a significant difference between Control and EtOH treated rats at P<0.05.

### Offspring Data

Offspring body weight was monitored and the weights at the beginning of behavioural testing are shown in Table 1. There were no significant differences in body weight between prenatal ethanol exposed (EtOH) and Control animals at 8mths of age or between Female Control and EtOH animals at 15 months of age. EtOH males weighed significantly less than Control males at 15 months of age (F(1,25) = 5.24, P<0.05).

### Behavioural Data

#### Elevated plus maze

**Figure 1:**
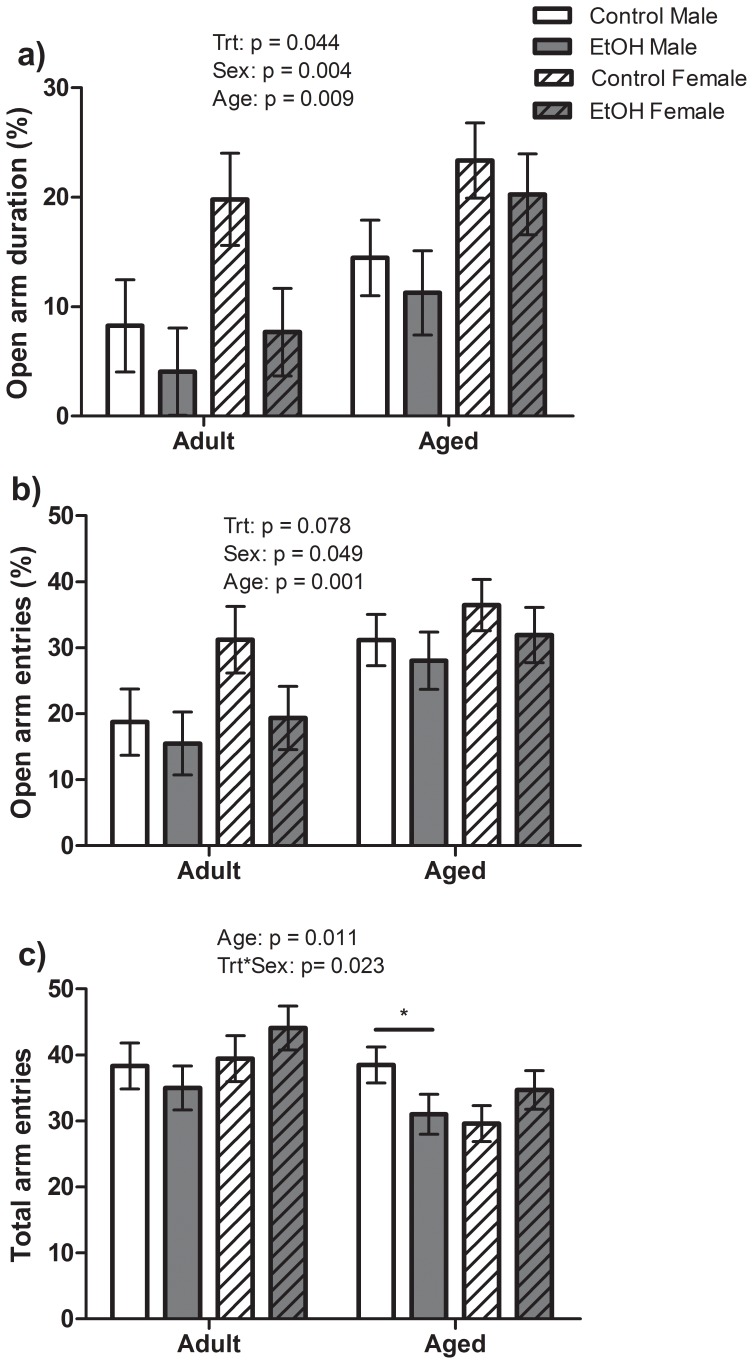
Anxiety like behaviour in the elevated plus maze. n = 9 Control male, 9 Control female, 10 EtOH male, 10 EtOH female Adult rats and 15 Control male, 15 Control female, 12 EtOH male and 13 EtOH female Aged rats included in the analysis. Statistical trends or significant effects determined by 3-way ANOVA are indicated by p values above each graph. a) Low dose prenatal alcohol exposed animals (EtOH) spent a significantly lower % time out on the open arms than their Control counterparts indicating increased anxiety-like behaviour. Female rats spent a significantly greater % time on the open arms than Male rats and Aged rats spent a significantly greater % time exploring the open arms than Adult rats. b) EtOH animals tended to have lower % entries into the open arms than Control animals. Female animals had significantly greater % entries than Male rats within each group and Aged animals had significantly greater % entries into the open arms than Adult animals. c) Adult animals made significantly more total arm entries (closed+open) than Aged animals indicating increased activity in Adult animals. No significant effect of Treatment or Sex was found to total number of arm entries. However, a significant interaction between Trt and Sex was found. Post hoc analysis of this interaction revealed a significant reduction in total arm entries for Aged Male EtOH animals compared with Aged Male Controls (indicated by line and star).

Open arm exploration and total arm entries in the EPM were assessed as an indication of anxiety-like behaviour and activity levels respectively. A significant main effect of Treatment (F(1,85) = 4.18, P<0.05) was found for open arm exploration in the EPM with offspring prenatally exposed to ethanol (EtOH) spending less time on the open arms of the maze (Figure 1a). In addition, a significant effect of Age (F(1,85) = 7.15, P<0.01) and Sex (F(1,85) = 8.92, P<0.01) was also found with Male rats spending less time on the open arms than Female rats and Aged rats spending a greater percentage time on the open arms than Adult rats. No significant interactions between these factors were found. A similar pattern was observed for the percentage open arm entries with significant effects of Age (F(1,85) = 11.20,P<0.01) and Sex (F(1,85) = 3.97, P<0.05) as well as a strong trend for prenatal treatment (F(1,85) = 3.18, P = 0.07) as shown in Figure 1b. A significant main effect of Age (F(1,85) = 6.79, P<0.05) was found for total arm changes during the plus maze trial with Adult animals making a greater number of total arm changes than Aged animals. A significant Treatment*Sex interaction (F(1,85) = 5.36, P<0.05) was also found for total arm changes although, there was no effect of Treatment or Sex alone (Figure 1c). Bonferroni post hoc analysis however, showed no significant differences between Control and EtOH animals for Adult or Aged, Male or Female rats.

#### Holeboard test

**Figure 2:**
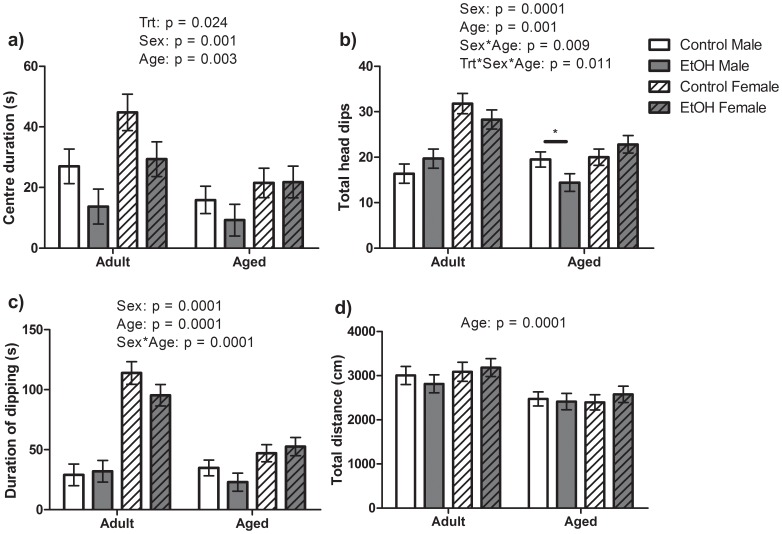
Measures of anxiety-like behaviour, neophobia and hyperactivity in the Holeboard test. n = 10 Control Male, 9 Control Female, 10 EtOH Male, 10 EtOH Female Adult rats and 15 Control Male, 15 Control Female, 12 EtOH Male and 12 EtOH Female Aged rats included in the analysis. Main effects as determined by 3-way ANOVA are indicated above each graph. a) Overall, prenatal ethanol treated rats (EtOH) spent significantly less time in the centre of the arena than Control animals indicating increased anxiety-like behaviour. Female animals spent more time in the centre of the arena than Male animals and Aged animals spent less time in the centre than Adult animals. b–c) Aged animals had reduced head dipping behaviour (number and duration) overall than Adult animals and Male animals had reduced head dipping behaviour than Female animals which may indicate an increase in neophobia in these animals. However an Age*Sex interaction was found for frequency and duration of head dipping and an Age*Sex*Trt interaction was found for the total number of head dips (b–c). Post hoc analysis of these interactions found Aged EtOH Males had significantly lower head dip frequency and duration than Aged Control Males (indicated by line and star). d)Aged animals travelled significantly less distance than Adult animals indicating increased activity levels in Adult animals.

The time spent in the centre of the holeboard arena, exploration of holes and total distance travelled were recorded as measures of anxiety-like behaviour, neophobia and activity respectively. A significant main effect Treatment (F(1,85) = 5.26, P<0.05) was found for time spent in the centre of the holeboard arena with EtOH rats spent significantly less time in the centre of the holeboard arena than Control treated animals (Figure 2a). Significant effects of Age (F(1,85) = 9.19, P<0.01) and sex (F(1,85) = 11.41, P<0.001) were also found with Adult rats spending more time in the centre of the arena than Aged animals regardless of Sex or Treatment group. Aged and Adult female rats in both treatment groups spent a greater amount of time in the centre of than male rats. No significant interactions between factors were found.

Significant effects of Age (F(1,85) = 11.91, P<0.001; 24.12, P<0.001) and Sex (F(1,85) = 34.14, P<0.001; 68.55, P<0.001) as well as significant Age*Sex (F(1,85) = 7.16, P<0.01; 21.42, P<0.001) interaction were also found for the frequency and duration of head dipping respectively. A significant Age*Sex*Treatment (F(1,85) = 6.81, P<0.05) interaction was also found for head dip frequency only. Aged Control male rats dipped more often and for a greater total amount of time than Adult Control males. Female rats head dipped more frequently and more time than male rats regardless of age or treatment group. Bonferroni post hoc analysis of head dipping behaviour found that Aged EtOH treated males head dipped significantly less (F(1,26) = 4.91, P<0.05) and tended to spend less time with their heads dipped (F(1,26) = 3.17, P = 0.08) than Aged Control males (Figure 2b–c). A significant effect of Age was found for total distance travelled in the arena with Adult animals travelling a significantly greater distance than Aged animals during the trials (F(1,85) = 17.11, P<0.001). However, no significant effect of Sex or Treatment group and no interaction between variables were found for this distance travelled.

#### Emergence test

**Figure 3:**
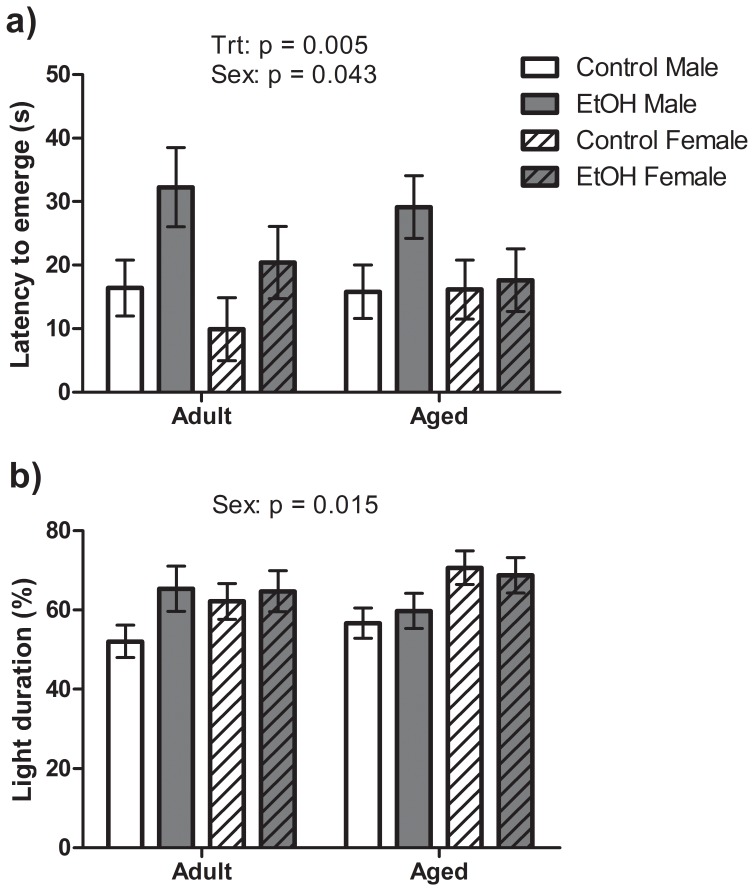
Anxiety like behaviour in a light dark emergence test. n = 10 Control Male, 9 Control Female, 8 EtOH Male, 8 EtOH Female Adult rats and 11 Control Male, 10 Control Female, 8 EtOH Male and 8 EtOH Female Aged rats included in analysis. Significant main effects as determined by 3-way ANOVA are indicated above each graph. a) Prenatal ethanol exposed (EtOH) animals had a significantly longer latency of first emergence than Control animals indicating increased anxiety-like behaviour. Additionally, Female rats had significantly lower latency of first emergence than Male rats. b) Male rats spent significantly less time outside the dark box than Female rats. No effect of Age was found on either measure in the emergence test.

Anxiety-like behaviour was also assessed in the light/dark emergence test by measuring the latency to emerge from the dark box and the percentage of time spent in the light area of the arena. A significant main effect of Treatment (F(1,65) = 8.36, P<0.01) was found for the emergence measure with EtOH animals displaying a greater latency to emerge than Control animals (Figure 3a). A significant effect of Sex (F(1,65) = 4.30, P<0.05) was also found with Male rats taking longer to emerge than Female rats. No significant effects of Age or interactions between factors were found for latency to emerge. No significant effects of Treatment or Age or interactions between factors were observed for the percentage time spent in the light area of the arena during the emergence trial (Figure 3b). However, a significant effect of Sex (F(1,65) = 6.28, P<0.05) was found with Female rats spending a greater percentage of time in the light area overall than Male rats.

#### Social interaction test

**Figure 4:**
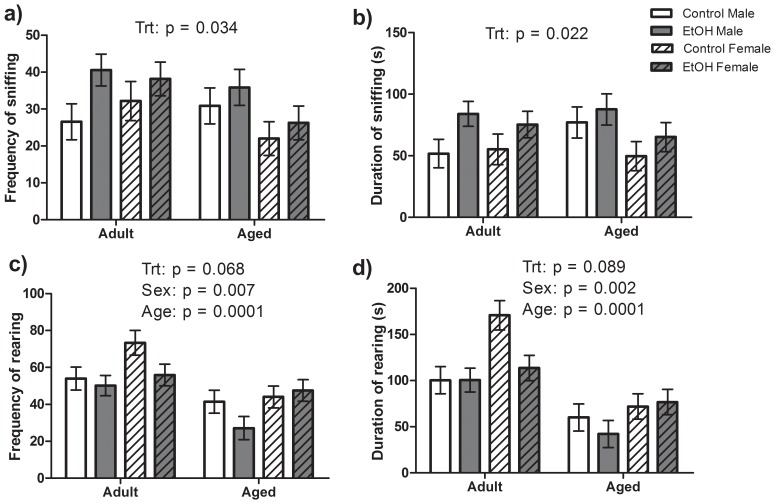
Exploratory behaviour in a Social Interaction Test. A total of n = 8 Adult Control, 9 Adult EtOH Male; 6 Adult Control, 7 Adult EtOH Female and 7 Aged Control, 7 Aged EtOH Male; 8 Aged Control, 8 Aged EtOH Females were used as “target” rats for the social interaction test. EtOH rats had increased frequency and duration of novel rat exploration (sniffing) than Control rats (Trt effect) (a–b). Increased novel arena exploration, frequency and duration of rearing, was found for Adult rats compared to Aged rats and in Female rats compared to Male rats (c–d).

A significant main effect of Treatment was found for the frequency (F(1,52) = 4.75, P<0.05) and duration (F(1,52) = 5.57, P<0.05) of novel rat exploration (sniffing) with EtOH investigating the novel rat more frequently and for longer than their control counterparts (Figure 4a–b). No significant effects of Age or Sex and no significant interactions between Age, Sex or Treatment on novel rat investigation. Significant effects of Age (F(1,52) = 18.26, P<0.001; F(1,52) = 33.71, P<0.001) and Sex (F(1,52) = 7.80, P<0.01; F(1,52) = 10.35, P<0.01) were found for the frequency and duration, respectively, of rearing behaviour (Figure 4c–d). Adult rats reared more frequently and for a greater total duration than Aged animals. Female rats reared more frequently and for a greater total duration than Male rats. There was also a tendency for EtOH rats to rear less frequently (F(1,52) = 3.48, P<0.07) and spend less time rearing (F(1,52) = 3.09, P = 0.08) than Control rats though this failed to reach statistical significance. No significant interactions of Age, Sex or Treatment were found for rearing behaviour.

### Histology

#### Stereology

**Figure 5:**
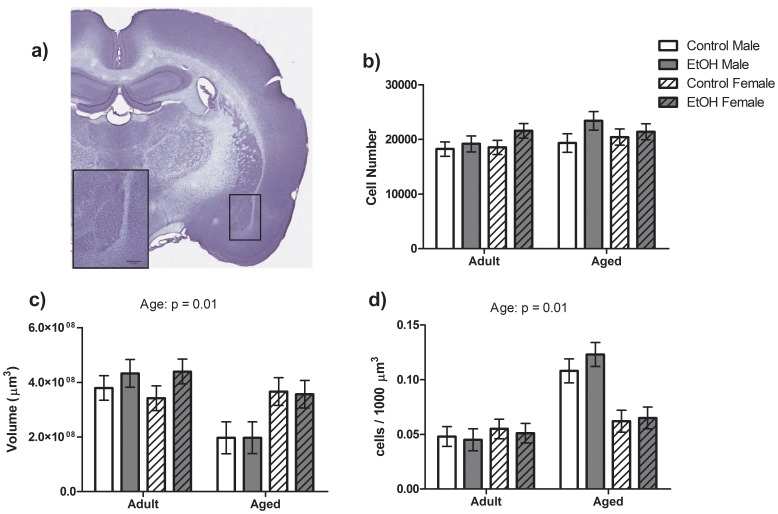
Stereological analysis of BLA Pyramidal Neurons. a) Nissl stained coronal section of a rat brain indicating outline of BLA. b) No significant differences were found for pyramidal cell number. Aged Male rats were found to have significantly smaller BLA volume (c) and a significantly greater pyramidal cell density (d). Prenatal treatment did not affect total volume or pyramidal cell density of the BLA.

No significant differences were found for total pyramidal cell number in the BLA (Figure 5b). However, a significant main effect of Age was observed for BLA volume and cell density with Aged Male animals having a smaller BLA volume (F(1, 20) = 35.80, P<0.01; Figure 5c) and a greater cell density within the BLA (F(1,20) = 31.97, P<0.01; Figure 5d) than Adult animals.

#### Apical dendrite morphology

**Figure 6:**
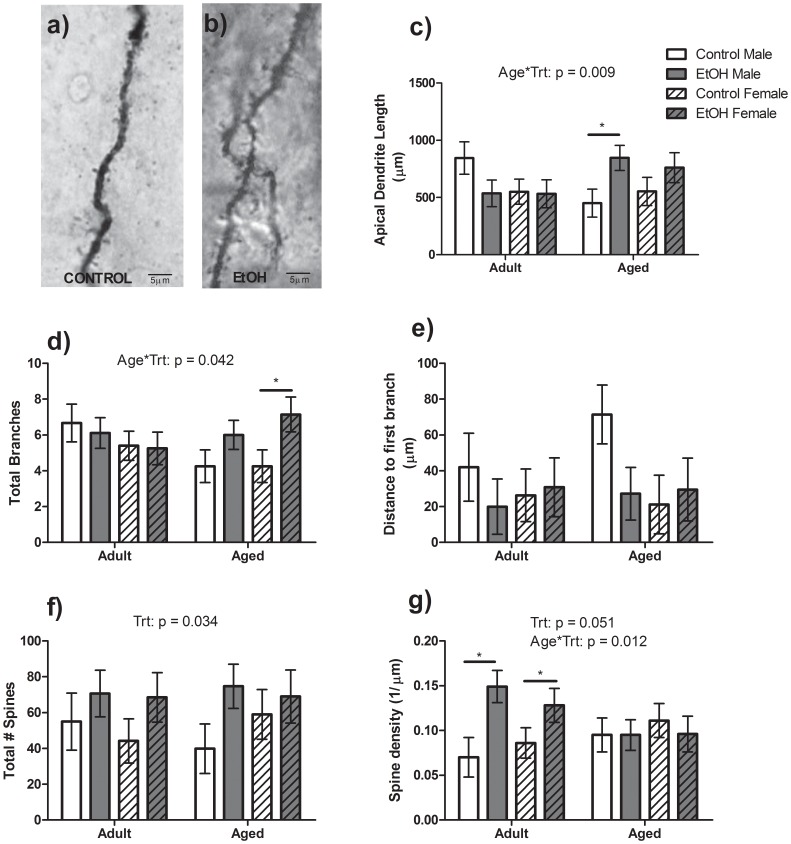
Apical dendrite morphology of Pyramidal Cells within the BLA. a–b) Apical dendrite section of a Control animal (a) and EtOH animal (b). Aged Male EtOH rats had a significantly greater total apical dendrite length than their Control counterparts (c). Aged EtOH Female rats had increased branching of the apical dendrite than their Control counterparts (d). No significant differences were observed in the distance to first branching point (e). EtOH animals had a greater number of spines present (f) and tended to have an overall increase in spine density along the apical dendrite of Pyramidal cells (g). A significant increase in spined density was observed in Adult EtOH animals compared to Adult Control animals.

No main effects of Age, Sex or Treatment were observed for total apical dendrite length or branch number of pyramidal cells in the BLA (Figure 6c–d). However, a significant Age*Treatment interaction (F(1, 40) = 7.24, P<0.01) was found and Bonferroni post-test revealed that Aged EtOH Male animals had greater total apical dendrite length than Aged Control Males (F(1,20) = 5.04, P<0.05), while Aged EtOH Female animals had greater branching of their pyramidal cell apical dendrites than Aged Control Females (F(1,20) = 6.96, P<0.05). There were no significant differences in the distance to first branch point (Figure 6e). A significant main effect of Treatment was found for total spine number along the apical dendrite with EtOH animals having a significantly greater number of spines than Control animals (F(1,40) = 4.71, P<0.05; Figure 6f). EtOH animals also tended to have a greater spine density (#spines/dendrite length) than Control animals though this did not reach statistical significance (F(1,40) = 3.95, P = 0.051; Figure 6e). However, a significant Age*Treatment interaction (F(1,40) = 6.69, P<0.01) was found for spine density with the post-test revealing a significant increase in spine density in Adult EtOH Male(F(1, 20) = 4.34, P<0.05) and Female (F(1,20) = 7.19, P<0.01) animals compared to Control animals of the same age.

**Figure 7:**
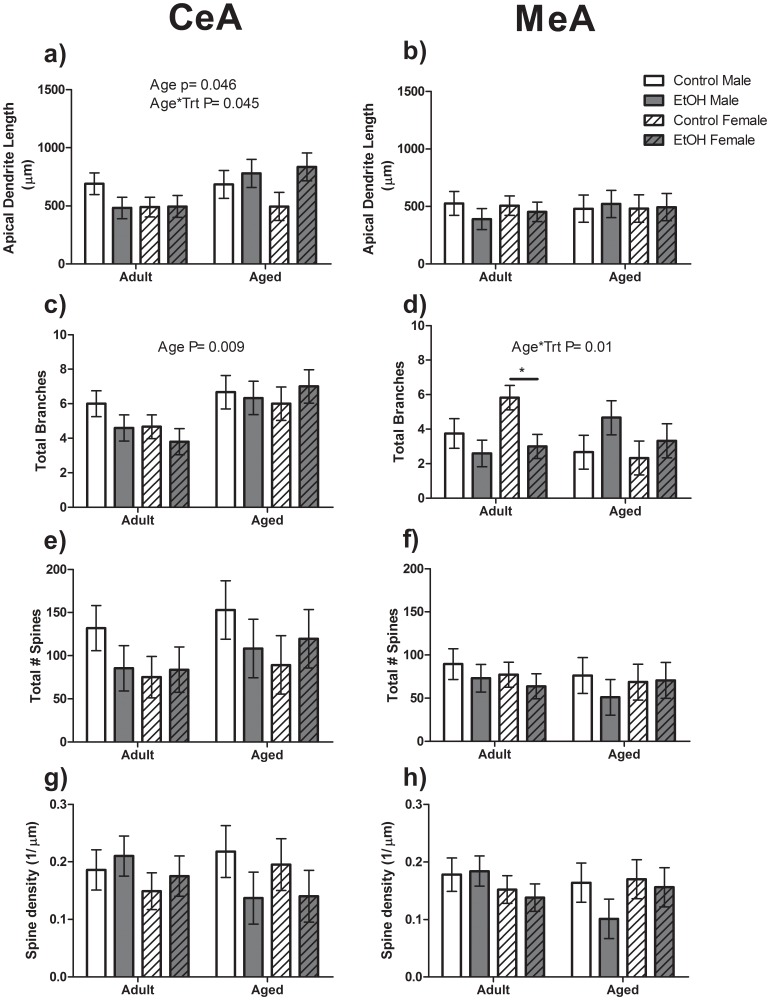
Apical dendrite morphology of Pyramidal Cells within the Central (CeA) and Medial (MeA) Amygdala. Aged animals averaged significantly longer apical dendrites (a) and greater number of branch points (c) than Adult animals in the CeA. There were no significant differences in apical dendrite length in the MeA (b). Adult Female Control animals had a significantly greater number of branch points than Adult Female EtOH animals (d, indicated by line and star). No significant differences in total number of spines or spine density along apical dendrites in either the CeA (e,f) or MeA (f,h).

To determine if these effects were specific to the BLA, the apical dendrite morphology of pyramidal cells was also assessed in the CeA and MeA of these rats. A significant effect of Age (F(1,40) = 4.43, P<0.05; Figure 7a) was found for apical dendrite length within the CeA with Aged animals on average having greater total apical dendrite length than Adult animals. A significant Age*Treatment interaction was also found for apical dendrite length in the CeA (F(1,40) = 4.45, P<0.05). However, Bonferroni post- test analysis showed no significant differences between groups. No effects of Age, Sex or Treatment were found for apical dendrite length in the MeA (Figure 7b). A significant effect of Age was also found for total number of branch points in the CeA (F(1,40) = 8.10, P<0.01; Figure 7c) with Aged animals having greater apical dendrite branching than Adult animals. A significant Age*Treatment interaction was found for the total number of branches in the MeA (F(1,40) = 7.84, P = 0.01; Figure 7d) with the Bonferroni post-test revealing that Adult EtOH Females had significantly fewer branch points than Control Females within the same age group (F(1,20) = 6.54, P<0.05). There were no significant differences in the CeA or MeA for total number of spines, spine density (Figure 7e–h) or the distance to first branch point (data not shown).

## Discussion

The aim of this study was to investigate the long term effects of low dose prenatal alcohol exposure on anxiety-like behaviour and the morphology of the BLA. Our low dose prenatal ethanol exposed animals exhibited a clear but subtle phenotype for anxiety-like behaviour in the EPM, holeboard, and emergence tests, which was present in both adult and aged animals. This increase in anxiety–like behaviour could not be attributed to a change in pyramidal cell number within the BLA but rather was associated with an increase in dendritic spines along the apical dendrite, found to be specific to the BLA, which may be indicative of an increase in synaptic connectivity and activity within these neurons [45], [46]. Interestingly, while there were some occurrences of sex specific effects of prenatal alcohol exposure on anxiety-like behaviour, these occurrences could not consistently be attributed to one sex. Aged animals exhibited a general decrease in overall activity during behavioural tasks that was not affected by prenatal treatment. However, prenatal ethanol exposure did have differential effects on dendritic morphology depending on animal age. Increased activity within the BLA was indicated in Aged ethanol exposed animals by greater length and branching of their apical dendrites whereas, in Adult ethanol exposed animals an increase in activity was indicated by a greater spine density along the apical dendrite. Taken together, this study highlights that consumption of even relatively modest amounts of alcohol during pregnancy has the potential to alter brain development and contribute to altered behaviour in adult and aged offspring.

The EPM, holeboard, and emergence tests are validated tests for anxiety related behaviours in rodents [41], [42], [47], [48]. Although, to our knowledge, no previous study has utilised the holeboard or emergence tests in prenatal alcohol exposure models, anxiety- like behaviour has been previously reported in the EPM and various other anxiety related measures after high dose prenatal alcohol exposure [26], [27], [29], [32], [49], [50], [51]. Our data supports findings from Dursun et al [27] and Zhou et al [51] who found increased anxiety-like behaviour in the EPM and open field tests, respectively, in young adult (PN80-85) offspring after prenatal exposure high doses of ethanol (6 g/kg, GD7–20). Unlike our study however, both Dursun et al [27] and Zhou et al [51] only tested male offspring. On the other hand, Hoffman et al [29] utilised feeding behaviour in a novel environment to investigate anxiety-like behaviour in both male and female offspring and found that prenatal exposure to a liquid diet containing 36% ethanol derived calories significantly increased the latency of feeding in female offspring only, indicating that prenatal alcohol induced anxiety like behaviour may be sex specific. However, similar to our results, Brocardo et al [32] did not find any interaction between effects of prenatal alcohol exposure in the elevated plus maze and the sex of the offspring. Instead, Brocardo et al [32] found that exposure to high doses of ethanol during gestation (4.3 g/kg, GD1–22) and the early postnatal period (4 g/kg, PN4–10) resulted in significantly decreased open arm exploration in the EPM in both male and female young adult (PN60) offspring.

In contrast to our study most of these previous studies conduct only a single test relating to anxiety-like behaviour and often only at one age. While this is a common method for investigating behavioural phenotypes, if used in isolation it is difficult to draw comprehensive conclusions regarding treatment effects on that particular phenotype. The use of multiple measures of anxiety-like behaviour in our study allows us to show persistent anxiety-like behaviour across tests and adds strength to our findings that low dose prenatal alcohol exposure leads to a subtle phenotype for anxiety-like behaviour. This is particularly beneficial because this is the first study to report anxiety-like behaviour after exposure to only relatively small amounts of alcohol *in utero*.

The results of the social interaction test are somewhat paradoxical when taken in relation to the phenotype present in the EPM, holeboard and emergence tests. Typically, a decrease in social interaction is associated with anxiety-like behaviour [52], [53] and our prenatal ethanol treated animals exhibit an increase in social interaction. However, unlike the social interaction paradigms reported within the literature which usually introduces singly housed unfamiliar rats into a familiar environment [53], [54], our paradigm consisted of exposure of group housed rats to a novel environment in conjunction with exposure to a novel rat. Thus, there was a conflict between the rat’s motivation to explore a novel conspecific and a novel environment. It may be that EtOH rats have a need for more social support in a novel environment than do control rats. Clearly the effects of prenatal alcohol exposure were dissociable on tests of anxiety (EPM, holeboard and emergence tests) and on social interaction.

While prenatal alcohol exposure has been shown to have detrimental effects on the structure and morphology of the hippocampus, cerebellum and some cortical regions [55], [56], [57], [58], [59], [60], [61], [62], [63], [64], [65], to our knowledge, no previous studies of prenatal alcohol exposure have looked at the structure or morphology of the amygdala. Interestingly we saw an increase in dendritic arborisation in the BLA which was not present in the CeA or MeA in prenatal alcohol exposed animals. Similar changes have often been associated with increases in neuronal activity within this region as well as increases in anxiety in models of acute stress, chronic stress and prenatal stress [66], [67], [68]. Though our study is the first to investigate the effect of prenatal alcohol exposure on the amygdala histologically, the increase in dendritic morphology and synaptic spine density along the apical dendrite of pyramidal neurons within the BLA is in line with a study conducted by Zhou *et al* [51]. Zhou and colleagues [51] showed hyperexcitability of neurons within the BLA, and anxiety-like behaviour in an open field test after prenatal exposure to relatively high amounts of alcohol in a rat model. However, it is interesting to note that studies examining anxiety-like behaviour and dendritic morphology within the amygdala after acute ethanol exposure in Adult rats have shown increased anxiety-like behaviour associated with increased dendritic spine density within the CeA and MeA with no differences in dendritic morphology in the BLA [69]. The mechanism by which prenatal alcohol exposure leads to increased activity in the BLA and altering anxiety and fear related behaviours is still unknown. The contrast in regional specific consequences of acute compared to prenatal alcohol exposure within the amygdala may suggest that the changes induced by prenatal alcohol exposure occur via downstream mechanisms.

Prenatal alcohol exposure has also been linked to hyperactivity of the stress response [49], [70]. Although we did not obtain measures associated with the stress response in our study, we would be remiss not to consider the possibility that hyperactivity of the stress response could, in part, be responsible for our neurological and behavioural findings given that dendritic arborisation in the BLA has also been shown in models of actute and chronic stress [66], [71], [72]. Suppression of the GABAergic system due to elevated glucocorticoid levels has also been shown to play a role in synaptic plasticity within the BLA [73], [74]. While we cannot draw any conclusions about the GABAergic system from our study, the study by Zhou *et al* [51] presents a strong case for the involvement of GABAergic interneurons in prenatal alcohol induced anxiety- like behaviour by showing that treatment with midolazam (MDZ), a positive modulator of the GABA_A_ receptor, effectively negated anxiety-like behaviour in prenatal EtOH exposed offspring. In addition to the behavioural response, Zhou et al [51] also showed that MDZ had minimal impact on control animals but effectively reduced excitability of neurons within the BLA of EtOH exposed offspring to a level analogous with controls. The inhibitory GABAergic system has been shown to be involved in the formation of fear and anxiety [75], [76]. Therefore, impaired function of the GABAergic system is an extremely plausible explanation for the underlying pathophysiology for anxiety-like behaviour induced by prenatal alcohol exposure.

Overall, our study successfully demonstrates that exposure to even a relatively small amount of alcohol during fetal development can result in an increase in synaptic connectivity specific to the BLA and induce a subtle anxiety-like behavioural phenotype that persists into ageing. The tasks that now remain are to investigate the age of onset of these anxiety-like behaviours as well as the pathophysiological mechanism behind these changes to work towards determining the best method of treatment for prenatal alcohol exposed individuals that suffer from anxiety related problems.
